# Nécrolyse épidermique liée à l'application cutanée d'une solution d'hydroxyde de potassium

**DOI:** 10.11604/pamj.2015.21.299.7673

**Published:** 2015-08-25

**Authors:** Nina Korsaga-Somé, Patrice Tapsoba, Muriel Sidnoma Ouédraogo, Léopold Baowendsom Ilboudo, Fatou Barro-Traoré, Pascal Niamba, Adama Traoré

**Affiliations:** 1 Service de Dermatologie-Vénéréologie, CHU Yalgado Ouédraogo, Université de Ouagadougou, Ouagadougou, Burkina Faso

**Keywords:** solution d´hydoxyde de potasse, nécrolyse épidermique, Ouagadougou, potassium hydroxide solution, epidermal necrolysis, Ouagadougou

## Abstract

Dans nos régions, une solution d'hydroxyde de potassium est régulièrement utilisée en application sur la peau comme traitement traditionnel dans le but de traiter un prurit ou une éruption cutanée. Dans notre pratique quotidienne, nous observons de plus en plus de réactions cutanées à ce type de traitement. Nous rapportons un cas de nécrolyse épidermique généralisée suite à l'application d'une solution concentrée d'hydroxyde de potassium. Il s'agissait d'une patiente de 51 ans, séropositive au VIH2, et au virus de l'hépatite virale C, mais non éligible au traitement antirétroviral. Elle était hospitalisée pour des décollements épidermiques nécrolytiques quasi-généralisés survenue le lendemain de l'application une solution de concentré de potasse sur tout le tégument. Cette solution avait été appliquée dans le but de traiter une éruption micropapuleuse prurigineuse généralisée (exanthème maculo-papuleux) survenue suite à a prise de cotrimoxazole. La réépidermisation était totale sans séquelle après un mois de suivi. Le diagnostic nécrolyse épidermique toxique ou syndrome de Lyell qui met souvent en jeu le pronostic vital avait été écarté devant la conservation de l'état général, l'absence d'atteinte muqueuse et la rapide cicatrisation. Certains traitements traditionnels ont fait la preuve au cours du temps de leur efficacité, mais un mésusage peut être à l'origine d'effets secondaires graves.

## Introduction

Les thérapeutiques locales sont souvent utilisées en automédication pour la prise en charge de certaines affections cutanées. Elles semblent moins dangereuses que les traitements généraux, mais elles peuvent entrainer parfois des effets secondaires caustiques ou allergiques. En effet, dans notre pratique quotidienne, nous observons de plus en plus de cas de réactions cutanées après application d'une solution d'hydroxyde de potassium sur le tégument, dans le but de traiter un prurit ou une éruption cutanée. Ces réactions vont d'une simple desquamtion à une nécrose cutanée. Nous rapportons un cas de nécrolyse cutanée suite à l'application d'une solution d'hydroxyde de potassium fortement dosée, sur une grande surface du tégument.

## Patient et observation

**Figure 1:**
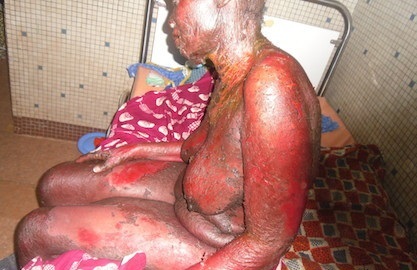
Décollement épidermique nécrotique généralisé. Source: collection service de Dermatologie-Vénéréologie du CHU-YO, Ouagadougou, Burkina Faso

Une femme de 51 ans, séropositive au Virus de l'Immunodéficience Humaine de type 2 (VIH2), et au virus de l'hépatite virale C, non traitée, était hospitalisée pour des décollements épidermiques nécrolytiques quasi-généralisés évoluant depuis deux semaines. L'examen notait un décollement épidermique nécrotique atteignant 98% de la surface corporelle, y compris le cuir chevelu, les paumes et les plantes sans intervalle de peau saine (Figure 1). Il n'y avait pas d'atteinte des muqueuses. L'état général était conservé. L'examen des autres appareils était sans particularité.

Deux semaines avant le début de cette symptomatologie, la patiente présentait une dysphagie traitée en automédication par du Cotrimoxazole. Deux jours après cette prise médicamenteuse, apparaissaient des lésions micropapuleuses prurigineuses généralisées à tout le tégument. Pour traiter cette éruption, une solution de concentré de potasse (deux phalangettes et demie de potasse diluées dans 250 cl d'eau) était appliquée sur tout le tégument. Le lendemain de ce traitement traditionnel, apparaissait une hyperpigmentation de tout le tégument avec une accentuation du prurit et un décollement cutané généralisé les jours suivants. L'état général était conservé.

**Figure 2:**
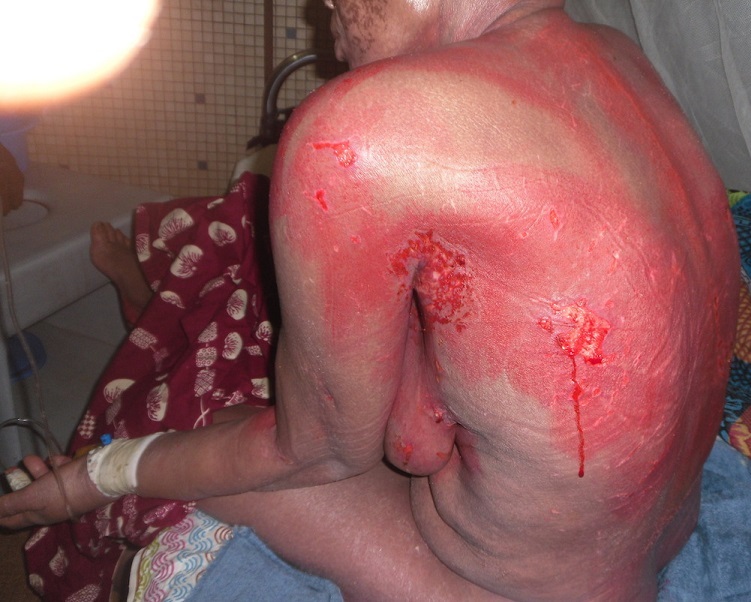
Epidermisation quasi-complète à J14 d'hospitalisation. Source: collection service de Dermatologie-Vénéréologie du CHU-YO, Ouagadougou, Burkina Faso

Les explorations paracliniques montraient une anémie microcytaire normochrome à 7,7g/100ml, une hypokaliémie à 3,1 mEq/l, (N=3,7 à 5,5), une augmentation des bicarbonates à 36,8 (N=22 à 29) et une hypoprotidémie à 54 g/l (N=65-80). Le taux de lymphocytes T CD4 s'élevait à 1503 cellules par microlitre. Des soins locaux faits de toilettes antiseptiques suivis de l'application d'émollients étaient administrés. L'évolution était marquée par une chute rapide des squames laissant des érosions très superficielles dès le 14^ème^ jour (Figure 2). La réépidermisation était totale sans séquelle après un mois de suivi.

Le diagnostic d'une toxidermie à type d'exanthème maculo-papuleux au Cotrimoxazole, compliqué d'une dermite caustique à type de nécrolyse épidermique exogène à la solution d'hydroxyde de potassium était posé. Ce diagnostic était retenu devant les arguments anamnestiques, le bon état général, l'aspect très superficiel du décollement, l'absence d'atteintes muqueuses et la rapide réépidermisation.

## Discussion

**Figure 3:**
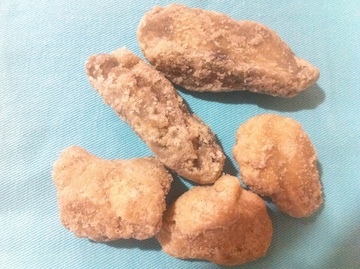
Blocs de potasse fabriqués traditionnellement. Source: collection service de Dermatologie-Vénéréologie du CHU-YO, Ouagadougou, Burkina Faso

La potasse est un minerais fait d'un mélange de carbonate de potassium et de chlorure de potassium; par extension, le terme potasse désigne les différentes solutions de sels de potassium et en particulier la solution aqueuse d´hydroxyde de potassium et la base KOH appelée aussi « potasse caustique ». Au Burkina Faso, la potasse est fabriquée de manière traditionnelle par les femmes. Elles font brûler du bois sur le sol, dans un lieu à l´abri du vent, pour obtenir un résidu de cendres qui est formé de sous-carbonate de potasse, de sulfate de potasse et de chlorure de potassium. Cette cendre est macérée dans de l'eau que l'on laisse évaporer afin d'obtenir un produit solide en bloc (Figure 3). Le mot potasse provient du néerlandais *«potas»* ou de l´anglais *«pot ash»* (littéralement « cendre de pot »). La potasse peut aussi être extraite sous forme de minerais dans des mines de potasse. Dans nos contrées, cettepotasse de fabrication traditionnelle se retrouve dans tous les foyers et est utilisée comme ingrédient dans la préparation des sauces (soit pour faciliter et accélérer la cuisson, soit pour réduire l'acidité d'un condiment) et dans la fabrication du savon traditionnel. Elle est aussi fréquemment utilisée en application sur la peau pour traiter un prurit ou une éruption cutanée. Lors de son usage à but thérapeutique en automédication, la potasse est diluée dans de l'eau et, selon le volume d'eau, le type de potasse et sa quantité, la concentration peut être très variée.

Si les effets bénéfiques des plantes médicinales sont reconnus et tendent à être de plus en plus valorisés, les risques liés à leur utilisation résident dans la difficulté à trouver le bon dosage. L'usage le plus courant est la décoction ou la poudre à ingérer et la poudre mélangée à un corps gras (souvent le beurre de karité) à appliquer sur la peau. Les effets secondaires des produits ingérés que nous rencontrons de plus en plus chez nos patients sont des atteintes hépatiques ou rénales souvent mortelles. Dans le cas présent le produit appliqué sur la peau avait été mal dosé. En effet, la concentration de la solution aqueuse d'hydroxyde de potassium a été estimée à 10%, expliquant l'importance de la nécrose épidermique. Des études réalisées chez le lapin ont montré que l'hydroxyde de potassium est un irritant cutané modéré suite à une exposition à des solutions < 5 %. Entre 5 et 10 %, les solutions d'hydroxyde de potassium sont sévèrement irritantes et deviennent corrosives au-delà de 10 % [1, 2].

Ailleurs, les dosages sont bien maîtrisés et les produits sont testés, avant l'utilisation. A Istanbul en Turquie et à São Paulo au Brésil, des équipes ont testé l'efficacité d'une solution aqueuse d'Hydroxyde de potassium (KOH) à diverses concentrations (2,5%, 5% et 10%), pour le traitement du molluscum contagiosum chez des enfants. Les solutions à 5% et 10% ont été les plus efficaces. Des effets secondaires à types de sensation de brûlures et d'hypopigmentation ont cependant été rapportés avec la solution à 10%. Les auteurs proposent néanmoins la solution aqueuse d'Hydroxyde de potassium à 5% ou 10% comme un traitement alternatif efficace, peu coûteux et non invasif du molluscum contagiosum chez les enfants [3–5].

En milieu professionnel, les principales voies d'exposition à la potasse sont les voies respiratoire et cutanée. La contamination cutanée ou oculaire entraîne localement des brûlures chimiques dont la gravité est fonction de la concentration de la solution, de l'importance de la contamination et de la durée du contact. Selon la profondeur de l'atteinte cutanée, on peut observer un érythème chaud et douloureux, la présence de phlyctènes ou une nécrose. L'évolution peut se compliquer de surinfection, de séquelles esthétiques ou fonctionnelles [6, 7].

Le diagnostic différentiel de cette nécrolyse épidermique exogène se discute avec la nécrolyse épidermique toxique ou syndrome de Lyell qui met souvent en jeu le pronostic vital. Nous avons écarté ce diagnostic devant la conservation de l'état général, l'absence d'atteinte muqueuse et la rapide cicatrisation. Nous pensons également que le terrain particulier de la patiente a pu jouer un rôle dans la violence de la réaction.

## Conclusion

Ce cas clinique nous interpelle sur 3 aspects: 1) La méconnaissance des produits utilizes; 2) La non-maîtrise de la préparation et du dosage de ces produits utilisés sans avis médical à l'origine de nombreux effets secondaires dont certains mettant en jeu le pronostic vital; 3) La confusion possible avec un syndrome de Lyell. Les patients doivent savoir que si certains traitements traditionnels ont fait la preuve au cours du temps de leur efficacité, un mésusage (mauvais dosage ou mauvaise utilisation) peut être à l'origine d'effets secondaires graves. Le traitement par hydroxyde de potassium ayant été proposé pour les molluscum contagiosa, des précautions d'emploi devraient être définies.
